# Unusual Triggers of Acute Intestinal Obstruction in Surgical Emergencies: A Series of Five Cases

**DOI:** 10.7759/cureus.60848

**Published:** 2024-05-22

**Authors:** Arun Singh, Shivani B Paruthy, Vaibhav Kuraria, Pramatheshwara S Aradhya

**Affiliations:** 1 General Surgery, Vardhman Mahavir Medical College and Safdarjung Hospital, New Delhi, IND; 2 General Surgery, Mysore Medical College and Research Institute, Mysore, IND

**Keywords:** small bowel obstruction, band obstruction, general emergency surgery, feeding jejunostomy, foriegn body ingestion, copper-t

## Abstract

Intestinal obstruction is a common surgical emergency that can be caused by mechanical causes or by different pathological processes. The most common cause of small bowel intestinal obstruction is post-operative adhesion, and the most common cause of large bowel obstruction is malignancy. These are classified into dynamic and adynamic types. The patient was selected based on the presentation management plan. Some cases require immediate operative intervention; however, some cases, as mentioned in this case series, require further investigation and a different approach. In this study, we report the rare causes of intestinal obstruction presented to Safdarjung Hospital, Department of General Surgery, New Delhi: one case of foreign body impaction, one case of spontaneous migration of feeding jejunostomy, one case of extrauterine IUCD causing intestinal obstruction, one case of mesentery band causing obstruction, and one case of abdominopelvic mass causing small bowel obstruction. These cases presented to the surgical emergency department with challenges in their diagnosis, intraoperative findings, and their outcomes.

## Introduction

Mechanical cause disrupts or impairs the forward movement of the intestinal contents, resulting in acute intestinal obstruction. Its prevalence among patients who visit the emergency room is estimated to be between 2% and 8% [1]. Although acute intestinal obstruction-related morbidity and mortality have decreased, clinical management remains difficult. Acute intestinal obstruction is most frequently caused by herniation, neoplasms, and adhesions. Small bowel obstruction (SBO) is most commonly caused by adhesions following previous abdominal surgery, accounting for 60% to 75% of cases. Adhesive SBO is more common after lower abdominal and pelvic surgeries such as appendiceal, colorectal, gynecological, and hernia procedures. Acute intestinal obstruction can lead to pathological repercussions, such as imbalances in fluid and electrolytes, as well as mechanical effects on intestinal perfusion due to increased luminal pressure [2]. Dehydration is caused by fluid loss due to vomiting, edema of the gut wall, and decreased absorptive capacity. Metabolic alkalosis results from the loss of gastrointestinal potassium, hydrogen, and chloride due to emesis. Severe dehydration promotes bicarbonate reabsorption and chloride loss in the renal proximal tubule, which maintains metabolic alkalosis [3].

Furthermore, stasis causes an overabundance of intestinal flora, which can result in bacterial translocation across the intestinal wall and the production of fecalization, a term for the formation of stool in the small intestine [4]. This procedure can occur quickly in a closed-loop obstruction, which is regarded as a surgical emergency, and involves a bowel segment that is clogged both proximally and distally. The classic closed-loop obstruction, intestinal volvulus, results in venous drainage and arterial inflow torsion, which immediately jeopardize intestinal viability [5]. Other causes of closed-loop obstruction include internal hernias, congenital bands, and intestinal malrotation [6]. Additionally, a functional ileocecal valve effectively converts an obstruction in the colon into a closed loop, which generally requires immediate decompression.

## Case presentation

Case 1

**Figure 1:**
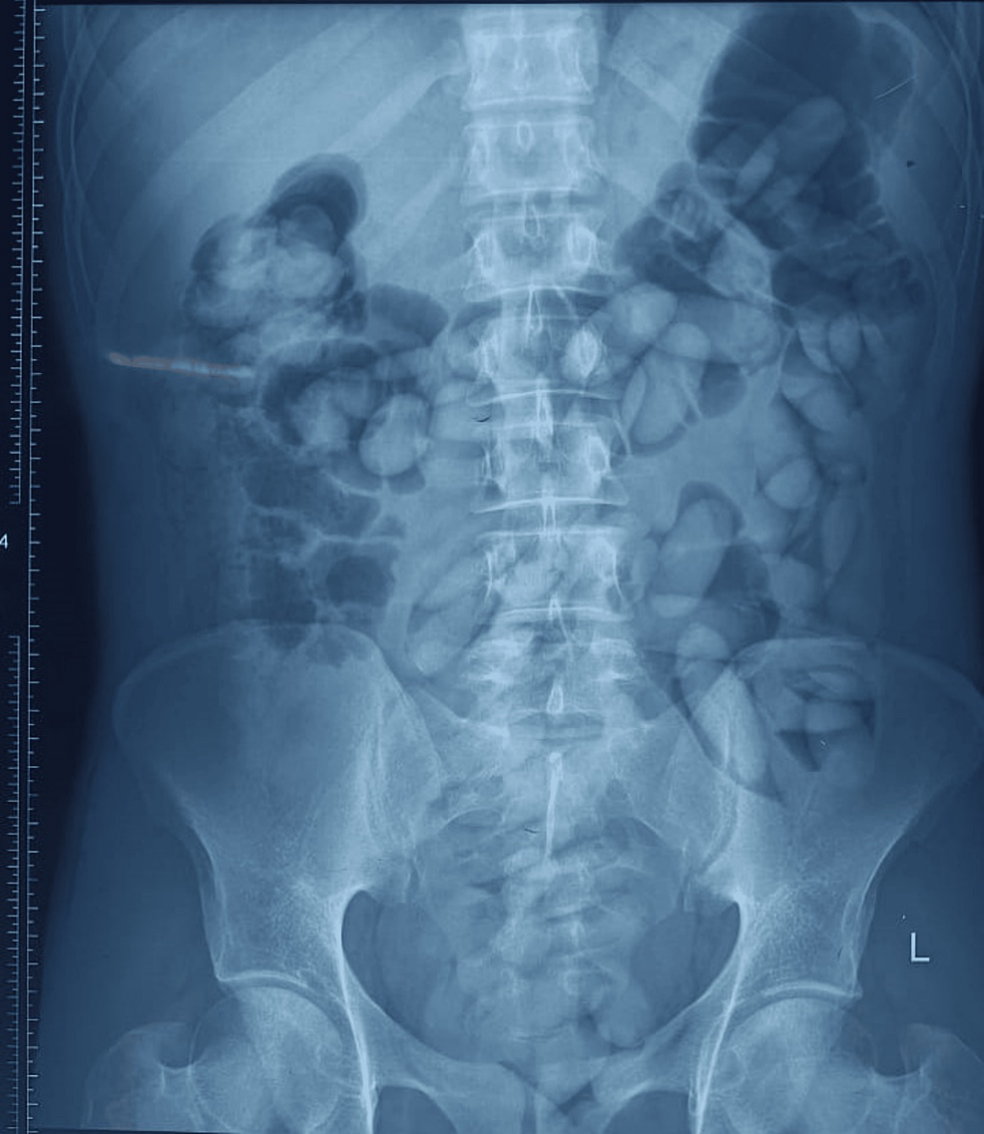
X-ray of the abdomen showing an impacted foreign body in the expected region of the large bowel and pelvis.

A 32-year-old male, a foreign national, with no known comorbidities, presented to the surgical emergency department with complaints of abdominal distension and non-passage of stool and flatus for one day. On physical examination, the patient was found to be vitally stable. The abdomen was distended, with tenderness over the left iliac and lumbar regions. The abdomen was soft on palpation, with no guarding or rigidity, and bowel sounds were raised. On a plain radiograph of the abdomen, multiple round-to-oval radiopaque structures were seen, reaching beyond the pelvic inlet in the expected region of the large bowel and rectum (Figure 1). Contrast-enhanced computed tomography (CECT) of the entire abdomen revealed multiple ovoid structures in the ascending, transverse, and descending colon, reaching the rectum. Based on radiological examination, the patient was diagnosed with intestinal obstruction due to multiple foreign body impactions. Expectant management was chosen as the treatment plan because there were no signs or symptoms of peritonitis. The patient was kept nil per oral (NPO) for the first 24 hours, after which a soft diet (including bananas) was allowed. The obstruction was relieved with spontaneous passage of foreign bodies in the stool. The hospital stay was uneventful, and the patient was discharged after radiological confirmation.

Case 2

**Figure 2:**
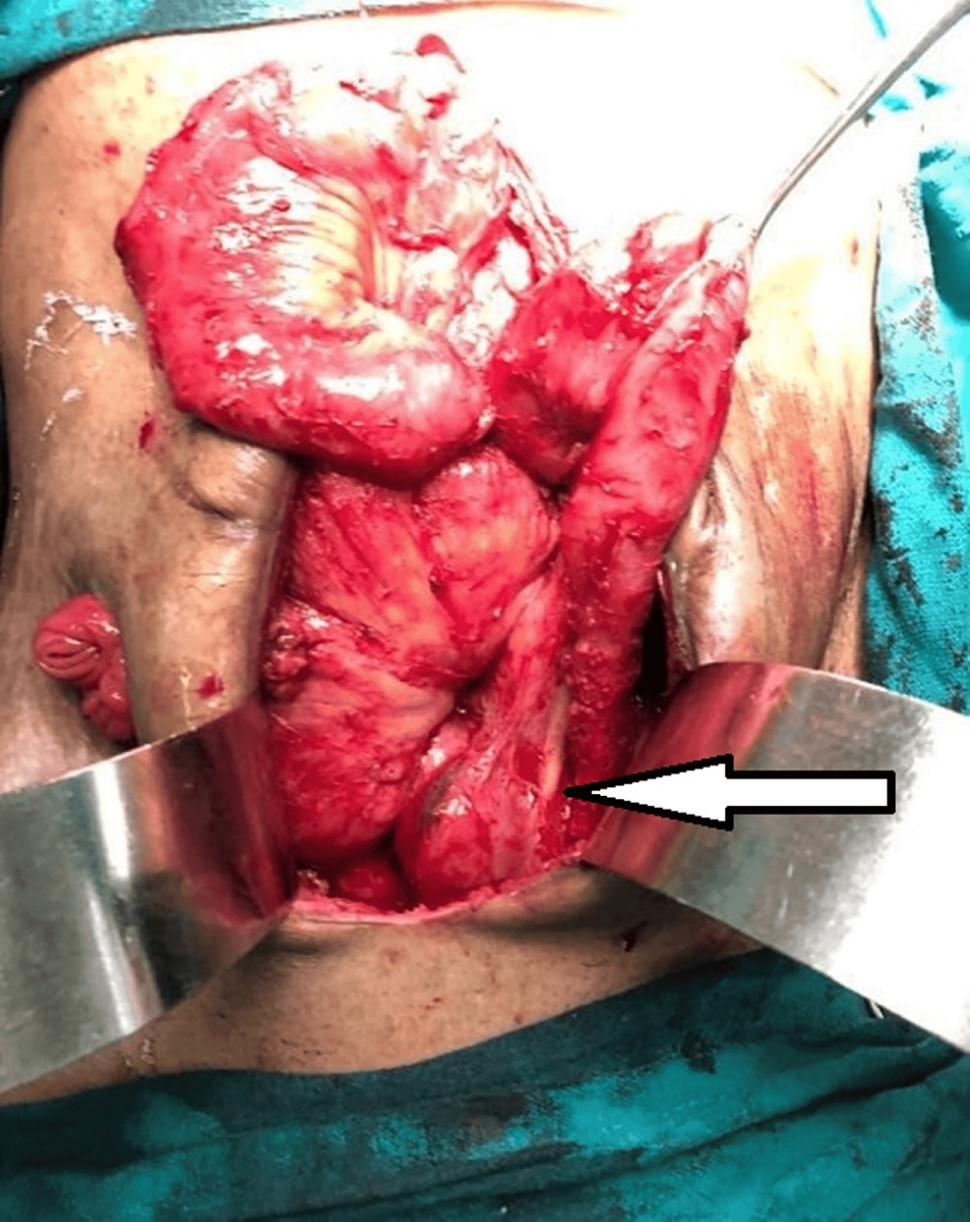
Intraoperative image of exploratory laparotomy with Foley's catheter visible in the small bowel (white arrow).

A 45-year-old male patient presented to the emergency department with abdominal pain, distension, and vomiting. On examination, the patient was tachycardic and pale. The abdomen was tense and distended with guarding and rigidity. Plain radiography of the abdomen revealed free air under the bilateral domes of the diaphragm. Perforation peritonitis was diagnosed. The patient underwent emergency exploratory laparotomy after initial resuscitation, during which two jejunal perforations were found 2.5 feet and 5.5 feet distal to the duodenojejunal (DJ) junction. The distal perforation was repaired. The proximal perforation was removed as a loop jejunostomy. Oral feeding was started on post-operative day (POD) 2. On POD 6, feeding from the distal stomal loop was started. The patient was discharged on POD 8 with a feeding tube (Foley’s catheter) in the distal loop of the jejunostomy, with advice on proper care of the tube. Two weeks later, the patient presented to the emergency with complaints of pain in the abdomen and missing/migration of a feeding tube in the distal limb of the bowel. The patient was tachycardic, hypotensive, and dehydrated. An abdominal examination revealed generalized tenderness. The proximal loop of the jejunostomy was functional. Initial resuscitation was performed. Endoscopic retrieval of the feeding tube was attempted but failed. The patient underwent exploratory laparotomy and retrieval of a foreign body (Foley’s catheter) with jejunostomy closure. A Foley catheter, which was removed from the distal jejunostomy opening by retrograde milking and jejunostomy closure, was found in the distal ileal loop (Figure 2). The post-operative period was uneventful, and the patient was discharged on POD 7.

Case 3

**Figure 3:**
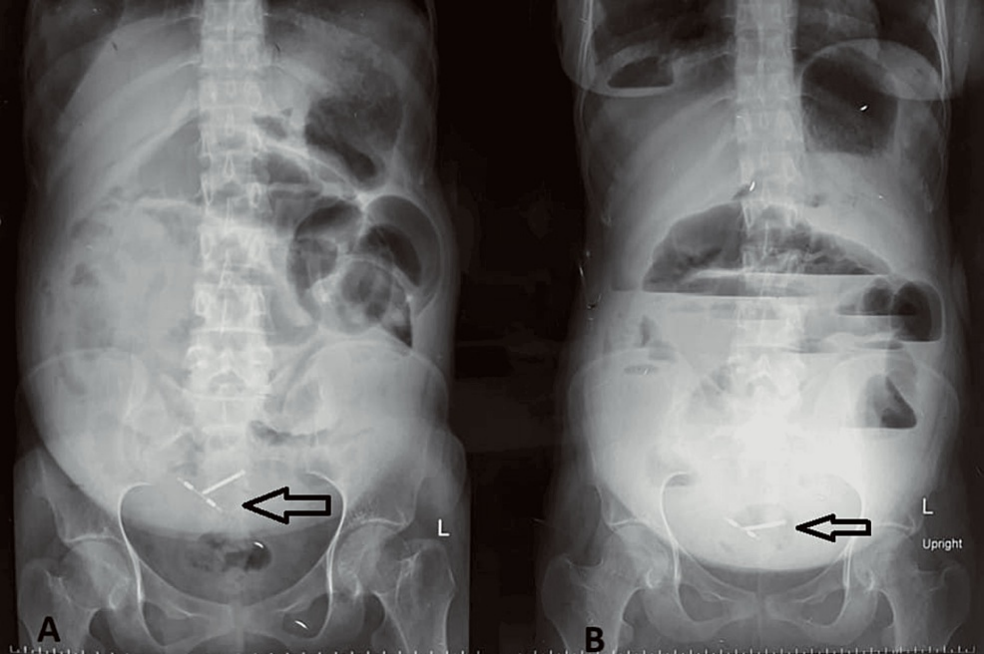
(A) Pre-operative plain radiograph of the abdomen showing copper T (black arrow) in the pelvis with multiple air-fluid levels. (B) Plain radiograph of the abdomen in supine position showing dilated bowel loops.

**Figure 4:**
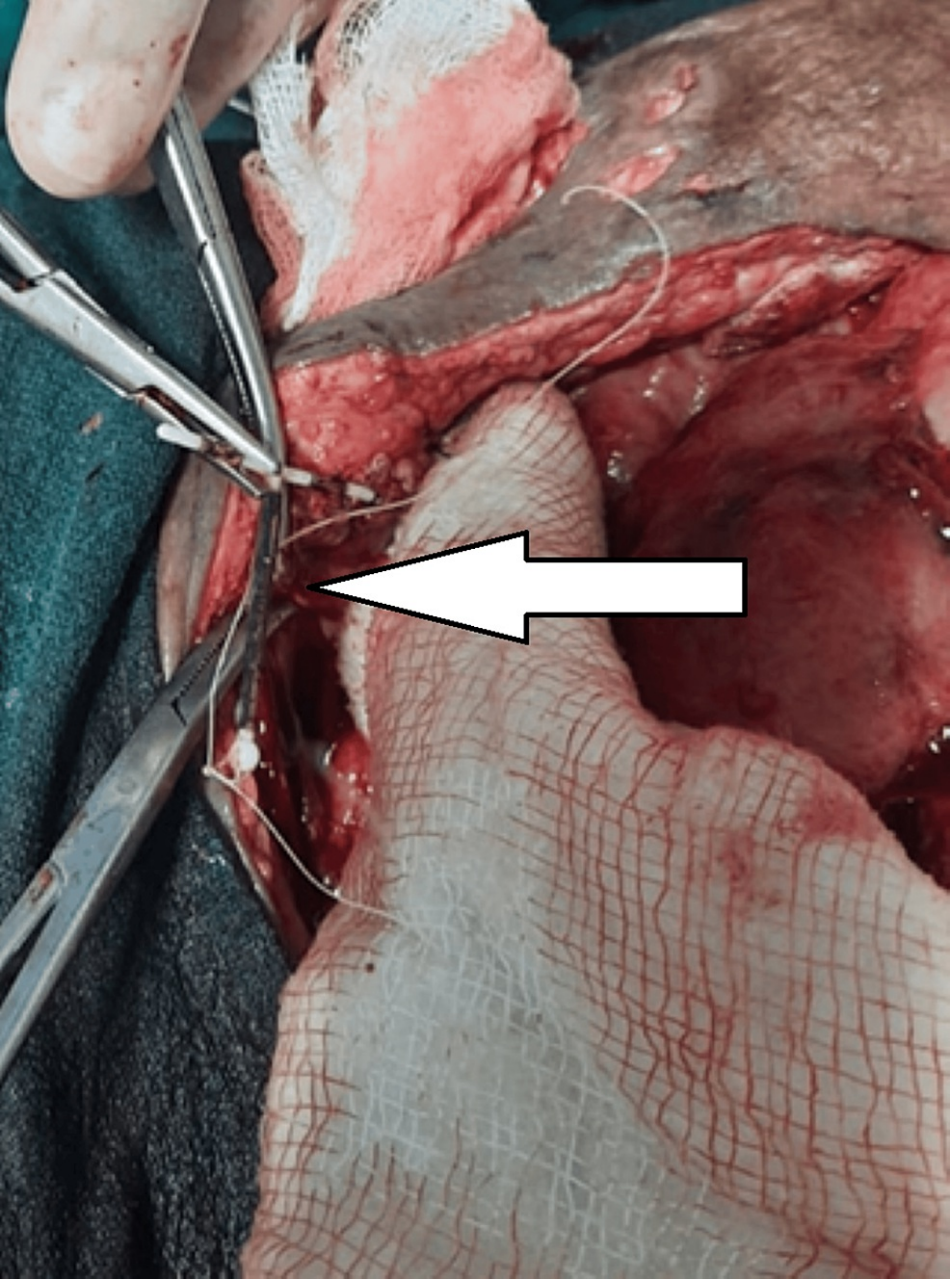
Intraoperative photograph showing free-floating copper T (white arrow) in the abdomen.

A 24-year-old female presented with abdominal pain and distension. The patient had a history of lower segment cesarean section one month prior to presentation. On physical examination, the abdomen was grossly distended with diffuse tenderness, bowel loops were standing out, and bowel sounds were absent. Digital rectal examination revealed no fecal staining on the gloved finger, and the rectal wall was dilated. The patient was stabilized and was sent for further investigation. On radiological examination, a plain X-ray of the abdomen showed multiple air-fluid levels with grossly dilated small bowel loops in the central abdomen and copper T visualized in the pelvis (Figure 3). Ultrasonography of the whole abdomen showed multiple dilated small bowel loops in the right and left iliac fossa with free fluid. The patient was diagnosed with an intestinal obstruction. The patient underwent emergency exploratory laparotomy after initial resuscitation, in which adhesiolysis with drainage of the purulent peritoneal collection was performed. On pelvic exploration, a free-floating copper T was found in the peritoneum, causing pyoperitoneum (Figure 4). No obvious perforations were observed in the uterus. After thorough peritoneal lavage, the patient’s abdomen was closed. The post-operative stay was uneventful, and the patient was discharged on POD 8.

Case 4

**Figure 5:**
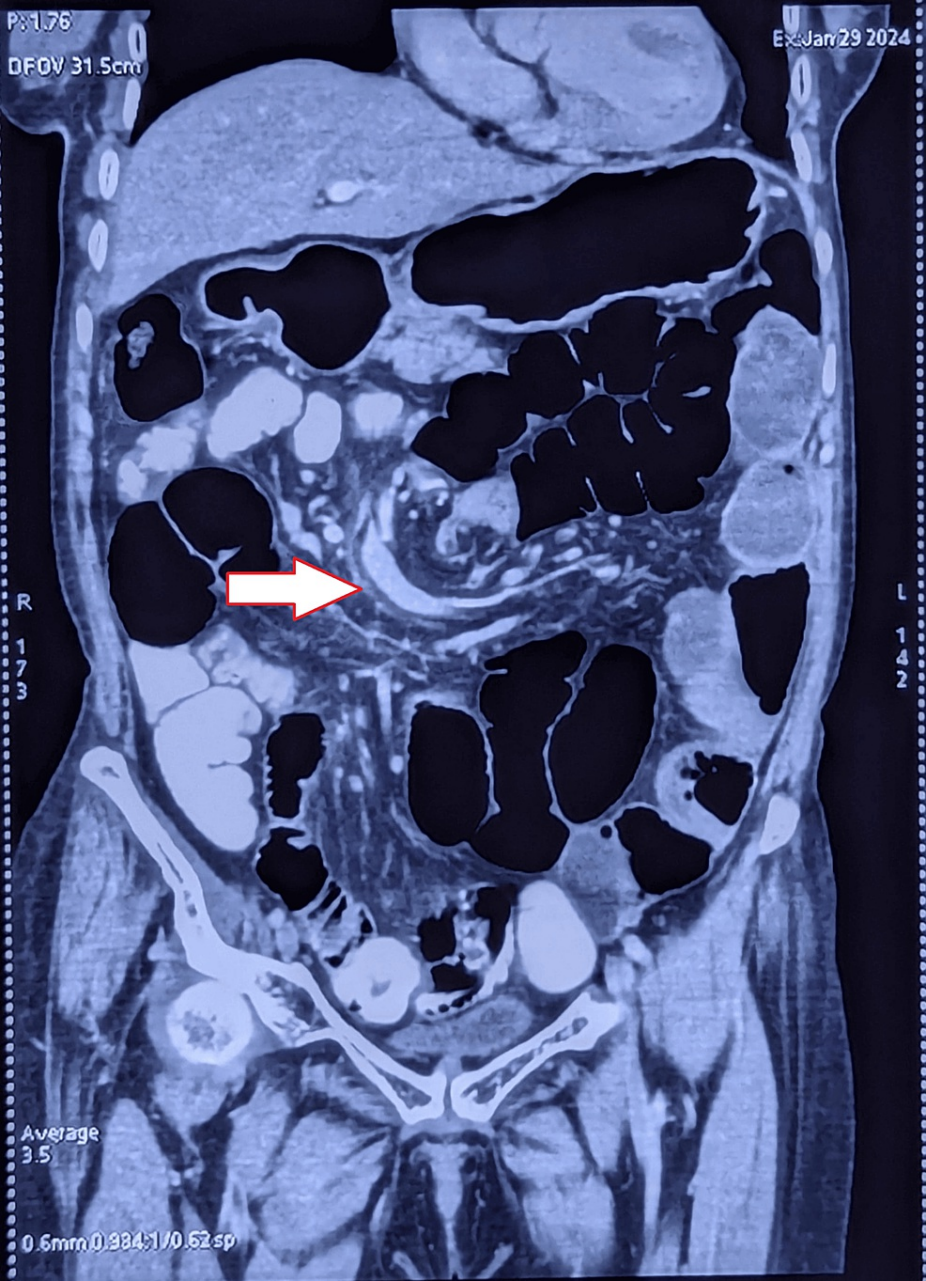
Contrast-enhanced CT of the whole abdomen showing mesenteric vessels with the swirl sign (white arrow).

**Figure 6:**
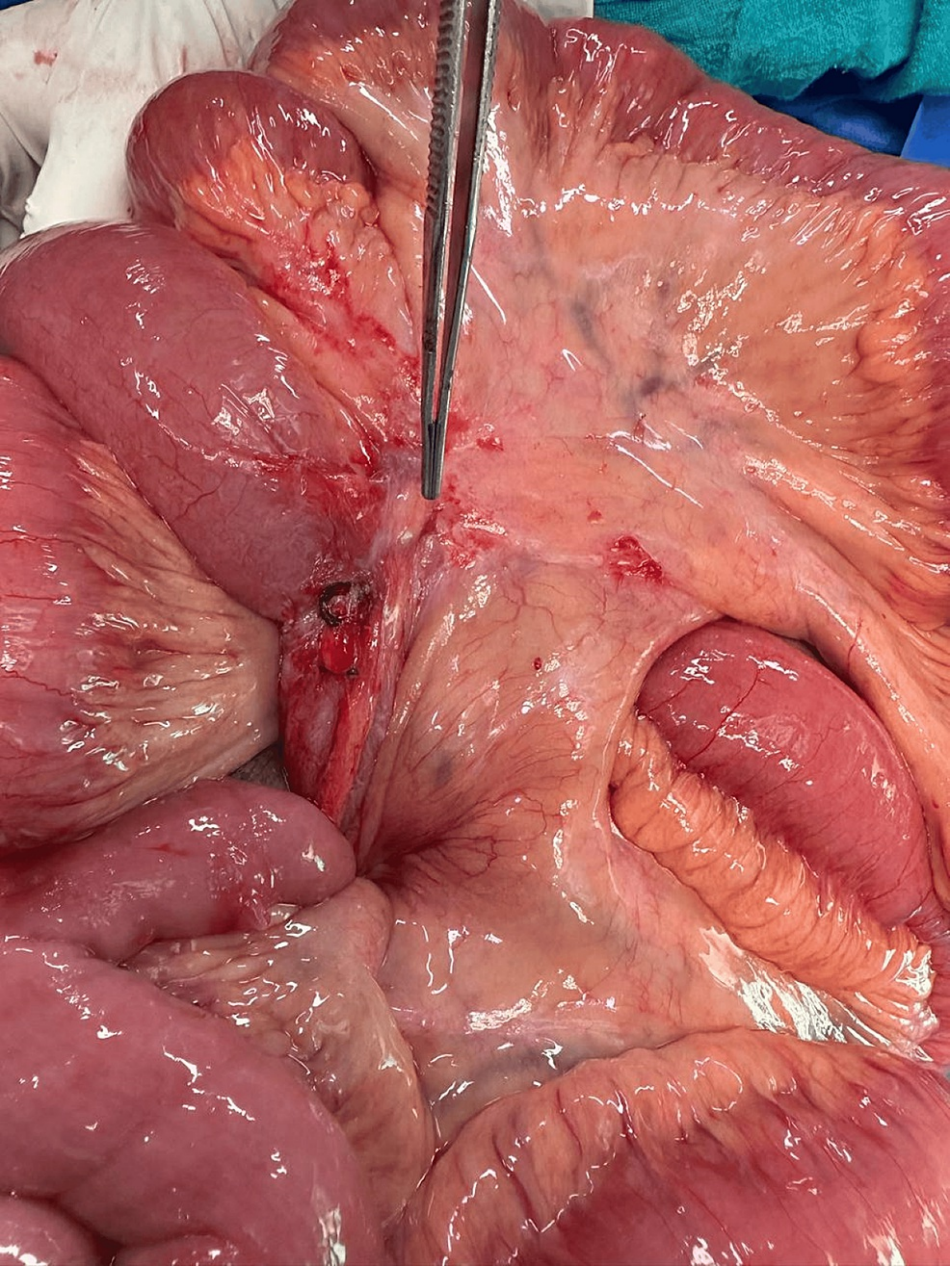
Intraoperative image showing a band causing small bowel obstruction.

A 66-year-old woman with a history of laparoscopic cholecystectomy one year ago presented to the surgical emergency department with complaints of pain in her right upper abdomen for one month and intermittent episodes of vomiting for one week. The pain was present in the upper abdomen, radiating to the back and aggravating on food intake, colicky in character with a history of constipation, and abdominal distension. On examination, the patient was vitally stable, and abdominal examination showed a distended abdomen with no guarding or rigidity but tenderness in the central abdomen. On digital rectal examination, anal tone was normal, fecal staining was present on the gloved finger, and the rectal walls were collapsed. Plain radiography of the abdomen revealed dilated bowel loops with air-fluid levels. On abdominal ultrasonography, the small bowel loop appeared to be dilated with mild free fluid in the pelvis. CECT revealed an abrupt transition at the terminal ileum with inter-bowel adhesions, and angulation of the bowel loop was noted at the adhesions. Swirling of the mesenteric vessels (Figure 5) was observed at the level of the adhesions, with multiple discrete mesenteric lymph nodes and loculated fluid collection. Adhesive intestinal obstruction was diagnosed, and the patient was taken up for emergency exploratory laparotomy. Intraoperatively, there was a thick band of 1 cm extending from the ascending colon to the descending colon, with internal herniation of a small bowel loop that was dusky in appearance and regained its normal appearance after 100% oxygen and warm saline. Five centimeters of the ileum proximal to the ileocecal junction was thickened and narrowed, and the bowel appeared dilated proximal to the band. A dilated sigmoid colon with a long mesocolon was observed (Figure 6). Adhesiolysis of the bowel with release of the band obstruction and plication of the sigmoid colon was performed. The patient was doing well post-operatively.

Case 5

**Figure 7:**
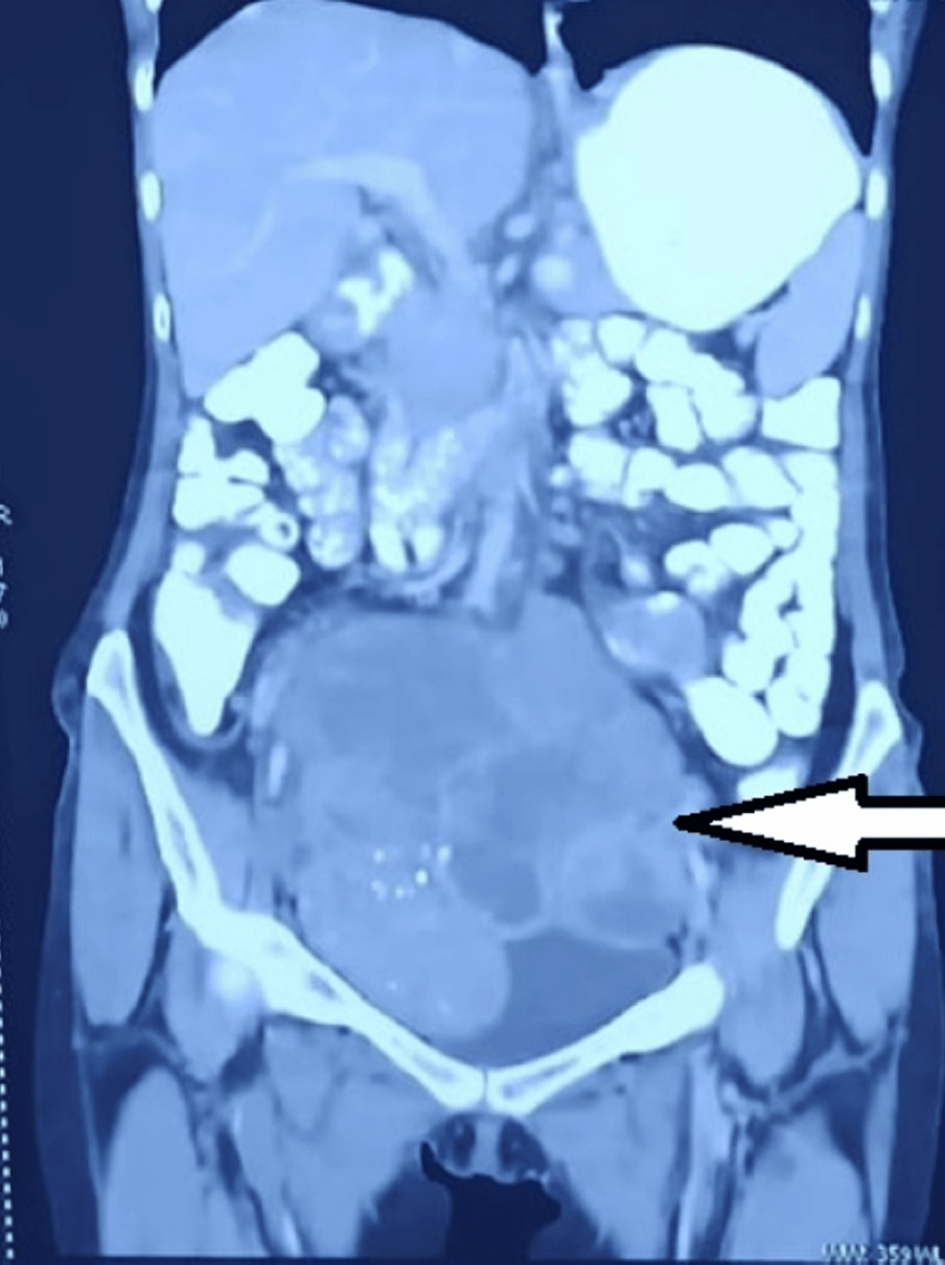
Pre-operative imaging (CT scan) showing a large abdominopelvic tumor (white arrow).

**Figure 8:**
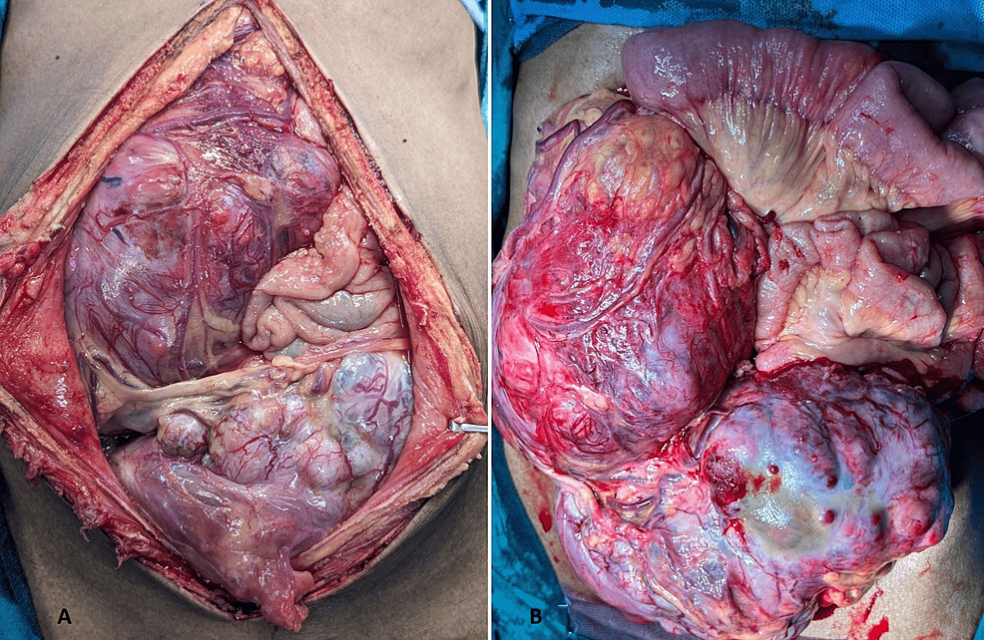
Intraoperative image of the tumor (30x18x16 cm) involving the whole abdomen, displacing the small bowel loops and causing obstruction. (A) Initial intra-abdominal extension of pelvic mass with displaced bowel loops in the left upper abdomen. (B) Delineated tumor mass with high vascularity.

A severely malnourished 37-year-old female presented to the emergency department with complaints of abdominal pain, multiple episodes of vomiting, and non-passage of stools and flatus for four days, as well as a lower abdominal lump for six months, for which she was followed up by the gynecology department. The patient attained menopause two years later. On examination, the patient was conscious and oriented with a pulse rate of 106 beats/minute and a blood pressure of 110/70 mmHg. Abdominal examination revealed a 30 × 20 cm hard palpable lower abdominal mass with immobility and tenderness, with smooth margins, and the lower border could not be palpated, with palpable bowel loops. The tumor marker CA-125 was also elevated (3164 U/mL). Ultrasonography showed a large abdominopelvic mass of size 32 × 24 cm almost reaching up to the epigastrium and filling the abdominal cavity, with few areas of cystic degeneration and internal vascularity, likely of ovarian origin. CECT showed a large heterogeneously enhancing partially calcified abdominopelvic space-occupying lesion likely to arise from the right adnexa with a mass effect on the sigmoid colon and bilateral ureters, features suggestive of a malignant mesenchymal tumor (Figure 7). The patient underwent emergency exploratory laparotomy. Intraoperatively, approximately 30 × 18 × 16 cm of a large multilocular, highly vascular abdominopelvic mass (Figure 8) likely arose from the adnexa involving the whole abdomen. Dense adhesions were present between the bowel, retroperitoneum, and uterus, and the tumor. Tumor infiltration was observed in the ileum for 10 cm, 5.5 feet distal to the DJ junction. This segment was resected, and a double barrel ileostomy was performed with en masse removal of the tumor with a total hysterectomy and bilateral salphingo-opherectomy. The patient was extubated and shifted to the high-dependency unit of the surgical ward. The stoma became functional on POD 3, and the patient was started on oral feeds (initially liquid diet followed by soft diet and full oral diet). Post-operative histopathological report of the resected mass suggested ovarian carcinosarcoma (malignant mixed Müllerian tumor) positive for vimentin, EMA, and p53, and negative for CEA and SALL4. The rest of the post-operative period was uneventful. The case was discussed on the tumor board, the patient was attached to the Medical Oncology Department for further management, and the patient was discharged on POD 7 and followed up on an outpatient basis.

Table 1 provides a consolidated list of all cases.

**Table 1:** Consolidated list of all cases

Patient	Age/sex	Diagnosis	Management
Case 1	32/male	Bowel obstruction in a body packer	Conservative
Case 2	45/male	Feeding jejunostomy tube as a traveler: migration of feeding tube from jejunostomy	Exploratory laparotomy and removal of the feeding tube
Case 3	24/female	Bowel obstruction with migrated copper T (an intrauterine contraceptive device)	Exploratory laparotomy with adhesiolysis with drainage of pyoperitoneum and removal of copper T
Case 4	66/female	Mesocolic band adhesion	Exploratory laparotomy with adhesiolysis
Case 5	37/female	Giant ovarian malignancy causing intestinal obstruction	Exploratory laparotomy with bilateral salphingo-opherectomy and total hysterectomy with en mass removal of tumor and double barrel ileostomy

## Discussion

Food impaction and foreign bodies are among the most frequent gastrointestinal ailments in emergency rooms. Foreign bodies are encountered in both adult and pediatric populations, and in 80%-90% of cases, they go away on their own without assistance. The job of a healthcare professional in case of foreign body is to identify patients who are at high risk of problems and need immediate attention [7]. In 2013, Jakhar et al. documented a case of a body packer who attempted to smuggle heroin by hiding it in his digestive system and died as a result. Autopsy verified that the body was retrieved within three to five days of the incident. Fifty pellets (packets) were found inside the body; two of the similar oval-shaped "egg" packages - six in the small intestine and two in the large intestine - were damaged [8]. In case 1, the patient was a foreign national brought by a customs officer from a New Delhi airport. The patient was vitally stable and showed no signs of distress; therefore, expectant management was chosen.

In a different case study published by Honar et al. [9], a 30-year-old man complaining of stomach ache was taken to the emergency room after being released from prison. Following the lack of signs of drunkenness and unremarkable plain abdominal radiographic findings, an abdominal computed tomography (CT) scan indicated that the patient had consumed packets in both the stomach and small intestine. Consequently, the patient's operation was decided immediately, and the medication packets were completely removed during the procedure. Three days after surgery, the patient was discharged in good general condition [9]. Unlike in our case, the patient was managed with surgical decompression.

Kundal et al. reported an unusual case of a 47-year-male with a history of a prepyloric perforation repair leak who presented on POD 14 with an enterocutaneous fistula and a feeding jejunostomy tube in situ [10]. The patient was evaluated and managed conservatively and discharged on enteral feeds, both orally and via a jejunostomy tube. One month after discharge, the patient presented with intestinal obstruction and a missing jejunostomy tube. Radiological investigations suggested enteral migration of the jejunostomy tube, which was managed conservatively, and the patient was discharged on day 3 of admission after rectal expulsion of the tube [10]. In case 2, the migrated feeding tube was coiled proximal to the ileocecal junction, causing acute intestinal obstruction, which warranted quick intervention to relieve the obstruction.

Intrauterine device (IUD) is one of the most widely used long-term reversible contraceptive methods worldwide, and perforation of the IUD is rare but one of the most serious complications; a 40-year-old woman who had an IUD (copper T) implanted one month after giving birth presented with temporary pelvic cramps and secondary amenorrhea seven months later, according to a case study by Nceboz et al. An eight-week pregnancy was discovered by the patient's ultrasonography and clinical findings, although laboratory results were normal. Transvaginal ultrasonography also showed that the IUD was linked to the colon, as it was situated outside the uterus close to the sigmoid colon. The patient chose to end the pregnancy, and diagnostic laparoscopy was performed at the same time, revealing intestinal perforation caused by IUD migration. Laparoscopy was used to remove the device, which was partially embedded in the sigmoid colon; nevertheless, laparotomy was required to open the colostomy because of bowel perforation [11]. In our case, the IUD was present in the peritoneal cavity, causing a foreign body reaction and pyoperitoneum, as no obvious perforation was found in the bowel.

Blockage caused by bands is uncommon in adults. Bands that lack clear acquired or embryological foundations are known as anomalous congenital bands (ACBs). A search of the literature for ACBs that cause large bowel obstruction showed only three cases: one involving a band between the ascending colon and the right lobe of the liver, another involving a congenital mesocolic band that obstructed the sigmoid colon, and a third involving a parietocolic band that blocked the descending colon [12]. Without surgery, inflammation of the epiploic appendage that has adhered to the abdominal wall or another intra-abdominal structure may be the cause of adhesive obstruction. The embryonic remains of the vitelloumbilical cord and mesourachus are unusual causes of adhesive obstruction [13]. In our case, the patient was a 66-year-old female with a one-month history of intermittent obstruction that was managed with laparotomy.

Ovarian carcinosarcoma, also called ovarian carcinosarcoma or malignant mixed mesodermal tumor of the ovary, is an extremely aggressive and uncommon pathological form of ovarian cancer that mostly affects the female reproductive system at the uterine location. Carcinosarcoma presents late, making pre-operative diagnosis difficult. The majority of patients receive a diagnosis while the cancer is progressing quickly. These cancers have a tendency to metastasize because early-stage symptoms and indications lack specificity and are challenging to identify. Abdominal mass, distension, discomfort, ascites, sporadic vaginal bleeding, and nonspecific gastrointestinal symptoms are the principal clinical signs in a few cases [14].

## Conclusions

Intestinal obstruction is a common surgical emergency, and clinicians should be aware of its rare causes and presentation. A thorough history and clinical examination are always the cornerstone of any case, and adjunct to clinical evaluation, radiological diagnosis is always helpful. Timely and correct treatment plans should be determined to alter disease progression and increase patient survival.
